# Childhood Trauma As a Mediator of the Association Between Autistic Traits and Psychotic Experiences: Evidence From the Avon Longitudinal Study of Parents and Children Cohort

**DOI:** 10.1093/schbul/sbac167

**Published:** 2022-11-26

**Authors:** Christina Dardani, Rik Schalbroeck, Paul Madley-Dowd, Hannah J Jones, Daniela Strelchuk, Gemma Hammerton, Jazz Croft, Sarah A Sullivan, Stan Zammit, Jean-Paul Selten, Dheeraj Rai

**Affiliations:** Population Health Sciences, Bristol Medical School, University of Bristol, Bristol, UK; Rivierduinen Institute for Mental Healthcare, Leiden, The Netherlands; School for Mental Health and Neuroscience, Department of Psychiatry and Neuropsychology, Maastricht University, Maastricht, The Netherlands; Section of Nuclear Medicine, Department of Radiology, Leiden University Medical Center, Leiden, The Netherlands; Population Health Sciences, Bristol Medical School, University of Bristol, Bristol, UK; Population Health Sciences, Bristol Medical School, University of Bristol, Bristol, UK; Medical Research Council Integrative Epidemiology Unit, Bristol Medical School, University of Bristol, Bristol, UK; National Institute for Health Research, Bristol Biomedical Research Centre, University Hospitals Bristol NHS Foundation Trust, University of Bristol, Bristol, UK; Population Health Sciences, Bristol Medical School, University of Bristol, Bristol, UK; Population Health Sciences, Bristol Medical School, University of Bristol, Bristol, UK; Medical Research Council Integrative Epidemiology Unit, Bristol Medical School, University of Bristol, Bristol, UK; Population Health Sciences, Bristol Medical School, University of Bristol, Bristol, UK; Population Health Sciences, Bristol Medical School, University of Bristol, Bristol, UK; National Institute for Health Research, Bristol Biomedical Research Centre, University Hospitals Bristol NHS Foundation Trust, University of Bristol, Bristol, UK; Population Health Sciences, Bristol Medical School, University of Bristol, Bristol, UK; National Institute for Health Research, Bristol Biomedical Research Centre, University Hospitals Bristol NHS Foundation Trust, University of Bristol, Bristol, UK; Division of Psychological Medicine and Clinical Neurosciences, Cardiff University, Cardiff, UK; Rivierduinen Institute for Mental Healthcare, Leiden, The Netherlands; School for Mental Health and Neuroscience, Department of Psychiatry and Neuropsychology, Maastricht University, Maastricht, The Netherlands; Population Health Sciences, Bristol Medical School, University of Bristol, Bristol, UK; National Institute for Health Research, Bristol Biomedical Research Centre, University Hospitals Bristol NHS Foundation Trust, University of Bristol, Bristol, UK; Avon and Wiltshire Partnership NHS Mental Health Trust, Bath, UK

**Keywords:** autism, psychosis, childhood trauma, polygenic risk

## Abstract

**Background:**

Little is known on whether associations between childhood autistic traits and psychotic experiences persist into adulthood and whether genetic confounding and childhood trauma influence them. Here we investigate the associations between childhood autistic traits and psychotic experiences until young adulthood and assess the influence of schizophrenia polygenic risk and childhood traumatic experiences, using the Avon Longitudinal Study of Parents and Children (ALSPAC) population-based birth cohort.

**Study design:**

We used a measure of broad autistic traits (autism factor mean score), and four dichotomised measures of autistic traits capturing social communication difficulties (age 7), repetitive behaviours (age 5), sociability (age 3), and pragmatic language (age 9). Psychotic experiences were assessed at ages 18 and 24 using the semi-structured Psychosis-Like Symptoms interview (PLIKSi). Traumatic experiences between ages 5 and 11 were assessed with questionnaires and interviews administered to children and parents at multiple ages.

**Study results:**

Broad autistic traits, as well as social communication difficulties, were associated with psychotic experiences that were distressing and/or frequent until age 24 (autism factor mean score, *n* = 3707: OR 1.19, 95%CI 1.01–1.39; social communication difficulties, *n* = 3384: OR 1.54, 95%CI 0.97–2.45). Childhood trauma mediated a substantial proportion of the identified associations (~28% and 36% respectively, maximum *n* = 3577). Schizophrenia polygenic risk did not appear to confound the associations. Multiple imputation analyses (maximum *n* = 13 105) yielded comparable results.

**Conclusions:**

Childhood trauma may be an important, potentially modifiable pathway between autistic features and later onset of psychotic psychopathology.

## Introduction

Autistic individuals are at an increased risk of developing a psychotic disorder.^1^ An increasing number of studies also indicate that subclinical psychotic experiences are more common in individuals with autistic traits in the general population. This association has been observed in cross-sectional studies^2,3^ and in studies that followed children with autistic traits to a maximum of age 18 years.^4–7^ Although psychotic experiences in adolescence are usually transient and of no clinical concern, persistent psychotic experiences have been associated with distress and poor mental health outcomes,^8,9^ including the development of psychotic disorder.^10^ Little is currently known on whether the associations between autistic traits and psychotic experiences persist into adulthood.

There is evidence suggesting a shared genetic basis between autism and psychotic disorders.^11^ For instance, several common and rare genetic variants have been found to be associated with both autism and psychotic disorder,^12^ genome-wide association studies (GWAS) have shown a strong genetic correlation between autism and schizophrenia,^13^ and common polygenic risk for autism has been associated with increased odds of psychotic experiences in the general population.^14^

However, the risk of psychosis in autism may also be influenced by environmental factors.^11^ A history of childhood trauma (in the form of exposure to abuse, neglect, and bullying) is one of the most consistently reported environmental risk factors for psychotic experiences and psychotic disorder.^15,16^ Socio-communicative differences may make individuals with autistic features vulnerable to childhood traumatic experiences, which may be exacerbated by reduced access to social support.^17–20^ Indeed, there is evidence that childhood maltreatment and/or bullying victimization is more common in autistic individuals,^21,22^ individuals with autistic traits,^23,24^ and individuals with higher autism polygenic risk scores (PRS).^25^

Few studies have examined whether childhood trauma influences the risk of psychosis in individuals with autistic traits. One study of college students found that a self-reported history of trauma did not explain the association between autistic and schizotypal traits,^23^ but the retrospective design was prone to recall bias and precluded causal inferences. A longitudinal study reported that adjusting for bullying victimization did not alter the association between childhood autistic traits and psychotic experiences, but formal mediation analysis was not conducted.^4^ In contrast, a recent longitudinal study reported that bullying victimization mediated the association between autistic traits and psychotic experiences in adolescents, although other traumatic experiences were not assessed.^26^ Therefore, it remains unclear whether and to what extent trauma mediates the association between autistic traits and psychotic experiences.

Using data from a UK population-based birth cohort, we examined (1) whether autistic traits assessed between ages 3 and 9 were associated with psychotic experiences measured at ages 18 and 24, (2) the extent to which any identified association was mediated by trauma experienced between ages 5 and 11, and (3) the possible confounding influence of several child and family factors including schizophrenia PRS.

## Methods

### Participants

We used data from the Avon Longitudinal Study of Parents and Children (ALSPAC), a population-based cohort study of children born to 14 541 pregnant mothers residing in the former county of Avon, United Kingdom, with an expected delivery date between April 1, 1991 and December 31, 1992. Of these pregnancies, there were 14 062 live births and 13 988 children who were alive at 1 year of age. When the oldest children were approximately 7 years of age, eligible samples who did not join the study initially were contacted, and additional participants were recruited. This resulted in a total of 15 454 pregnancies and 15 589 fetuses, of which 14 901 were alive at 1 year of age. Depending on the analysis conducted, we restricted our sample to participants with complete data on autistic traits, traumatic experiences, psychotic experiences, confounders, and/or schizophrenia PRS (Supplementary Figure 1).

Further information on the ALSPAC cohort is available on the ALSPAC website (http://www.bristol.ac.uk/alspac) and elsewhere.^27,28^ The study website contains details of all the data that is available through a fully searchable data dictionary and variable search tool (http://www.bristol.ac.uk/alspac/researchers/our-data/). Some data were collected using REDCap.^29,30^

Ethical approval for the study was obtained from the ALSPAC Ethics and Law Committee and the Local Research Ethics Committees. Informed consent for the use of data collected via questionnaires and clinics was obtained from participants following the recommendations of the ALSPAC Ethics and Law Committee at the time.

### Measures

#### Autistic Traits

 In accordance with previous studies in the ALSPAC cohort,^31^ we used a measure of broad autistic traits, estimated as the mean score of seven factors identified in a previous factor analysis of 93 available measures related to autism. Additionally, we used four measures of autistic traits, which were independent predictors of an autism diagnosis. These included social communication difficulties assessed with the Social Communication Disorder Checklist (SCDC) at age 7 years,^32^ difficulties in pragmatic language use assessed with the coherence subscale of the Children’s Communication Checklist at age 9 years,^33^ sociability assessed with a subscale of the Emotionality, Activity and Sociability Temperament Scale at age 3 years,^34^ and repetitive behavior assessed with measures obtained from the Development and Well-Being Assessment at age 5 years.^35^ Participants who had scores within the approximately highest 10% of the measure distribution were classified as being “case positive” for the autistic trait.^36^

#### Psychotic Experiences

 Psychotic experiences were assessed at ages 18 and 24 using the semistructured Psychosis-Like Symptoms interview (PLIKSi), administered by trained psychologists, and scored according to criteria predefined by the World Health Organization.^37^ The PLIKSi consists of 12 core questions covering hallucinations, delusions, and thought interference. Participants were asked about experiences that had occurred since age 12 years. Psychotic experiences were considered present if, at ages 18 and/or 24 years, one or more of the experiences was rated by the interviewer as suspected or definitely present, and if this was not attributable to falling asleep or waking up, fever, or substance use. We additionally examined psychotic experiences that had been distressing and/or frequent, since these experiences are more clinically relevant and predictive of psychotic disorder.^38^ Moreover, in subsequent sensitivity analyses we excluded reports of tactile hallucinations, which might be difficult to distinguish from the heightened tactile perception often seen in autism.^39^

#### Childhood Trauma

 The measures of childhood trauma and their associations with psychotic experiences have been described in detail elsewhere.^16^ In brief, we used a measure of childhood trauma between ages 5 and 11 based on responses to 57 questions from questionnaires and interviews about domestic violence (regular acts of physical violence taking place in the home), physical abuse (physical harm to the participant from caregivers or other adults), emotional abuse (emotional cruelty to the participant from caregivers or other adults), emotional neglect (caregivers not taking an interest in the participant’s life), sexual abuse (adults or older children forcing the participant into sexual activity, including attempts to do so), and bullying victimization (regular name-calling, blackmail, or assault by peers). Measures of sexual, physical, and emotional abuse, assessed contemporaneously by the participant and their caregivers between participant ages 5 to 11, were supplemented with data from a participant-completed questionnaire at age 22, as all data on sexual abuse, and most data on physical and emotional abuse prior to age 11, were based on parental report. Each type of trauma was coded as present or not, and a single trauma variable was created representing exposure to any type of trauma.^16^

#### Confounders

 Confounders were considered on the basis of existing evidence suggesting associations with autistic traits, traumatic events, and psychotic experiences.^24,27,40^ These included child sex (male/female), maternal parity (≤1 child versus ≥ 2 children), major financial problems in the family when the child was 8 months old (yes/no), maternal highest educational attainment (32 weeks gestation), maternal age (at delivery), maternal Crown-Crisp anxiety scores^41^ (18 weeks gestation), maternal depression measured with the Edinburgh Postnatal Depression Scale^42^ (EPDS; 18 weeks gestation scores ≥ 13), and child IQ scores at age 8 assessed with the Wechsler Intelligence Scale for Children third edition^43^ (WISC-III). In mediation analyses, four assumptions are made with respect to confounding. These include no unmeasured confounders for any of the paths and no measured or unmeasured confounder for the association between mediator and outcome which lies on the causal pathway from the exposure. In the current analyses, the above confounders were assumed to potentially confound all paths.

We also examined the potential confounding role of schizophrenia PRS. In children with available genotype data in ALSPAC, we calculated schizophrenia PRS using GWAS summary data for schizophrenia^44^ as our discovery sample (details available in Supplementary Methods 1). We used scores corresponding to a .05 *P*-value threshold, as it has been found to optimally capture schizophrenia liability across different samples.^44^

### Statistical Analyses

Statistical analyses were conducted in STATA/MP version 15. We compared individuals with and without autistic traits on confounder data, traumatic experiences, and psychotic experiences using Pearson χ^2^-test, independent-samples *t*-tests, and logistic regression analyses.

Using logistic regression, we estimated odds ratios (ORs) and 95% confidence intervals (95% CIs) for the associations between the five measures of autistic traits in childhood and psychotic experiences in young adulthood. We performed crude models and confounder-adjusted analyses, including a separate analysis adjusting for schizophrenia PRS in the sample with available genotype data.

**Fig. 1.**
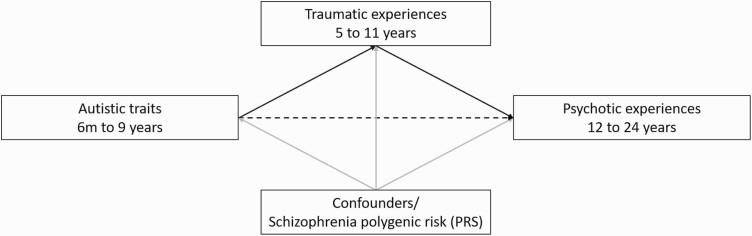
Schematic depiction of the mediation analyses, indicating potential indirect effects between exposure (autistic traits) and outcome (psychotic experiences) (solid black lines), potential direct effects (dashed black line), and potential confounding (grey lines). Although exposure and mediators overlap, the rationale of the analyses was based on the neurodevelopmental origins of autistic traits, ie that they are present since birth, regardless of assessment age. This is supported by previous studies in the ALSPAC cohort, suggesting associations between autism polygenic risk and the autistic measures used in the present analyses.^24,61^

Mediation analyses were performed in cases that there was evidence of association between the exposure(s) of the interest and the outcomes. Mediation analyses were performed using the g-formula package in STATA.^45^ We used the parametric g-formula using Monte Carlo simulations to estimate the natural direct effect (NDE) of autistic traits on psychotic experiences, and the natural indirect effect (NIE) that was mediated via traumatic experiences between ages 5 and 11. We performed unadjusted as well as adjusted models for confounders and for schizophrenia PRS (figure 1). Corresponding 95% CIs were estimated using the standard errors from 1000 nonparametric bootstrap resamples. The proportion mediated (PM) was calculated as^46^: [(OR_NDE_*(OR_NIE_ − 1))/ (OR_NDE_*OR_NIE_ − 1)]*100.

### Missing Data

We performed multiple imputation by chained equations,^47^ using the STATA *ice* command. Confounder, mediator, and outcome data were imputed for the sample with complete data on each autistic trait exposure. Provided the missing at random (MAR) assumption is met, multiple imputation (MI) can produce unbiased estimates even when the proportion of missing data is large. Specifically, previous work using simulations, found that for data with a MAR data pattern, multiple imputation can provide unbiased estimates even when the proportion of missing data is as high as 90%.^48^ We created 100 imputed datasets using information from variables included in our analyses as well as auxiliary variables associated with the variables of interest and attrition, to make the MAR assumption plausible.^49^ Based on established guidelines on auxiliary variables selection,^50^ we entered in the models those variables presenting the lowest missingness in the ALSPAC cohort ranging from 13% to 16% (Supplementary Methods 2). We used linear regression models for imputation of normally distributed variables, logistic regression models for binary variables, and the inbuilt *match* command for predictive mean matching to impute nonnormal continuously distributed variables. Considering that the MAR assumption is not directly testable, in the context of the present study we conducted a sensitivity analysis in cases that there was evidence of association between an exposure of interest and our primary outcome, psychotic experiences assessed at ages 18 and/or 24. Specifically, we assumed that all participants with missing data on the primary outcome variable, presented the outcome (ie, psychotic experiences at ages 18 and/or 24). We imputed covariates following the process described above and we ran crude and adjusted for covariates logistic regression models to test the association between the exposures and the outcome. This allowed us to scrutinize the association estimates across complete case, imputed data, and under the scenario that the MAR assumption was completely violated.

In the case of mediation analyses, we used the inbuilt g-formula imputation commands,^45^ allowing simultaneous imputation of missing data and mediation analyses, entering in the models the same auxiliary variables we used for the association analyses.

## Results

### Sample Characteristics

The maximum available sample size before imputation was 3707 for the analyses examining the associations between autistic traits and psychotic experiences, and 3577 for the mediation analyses (Supplementary Figure 1). Children scoring highest on all the autistic traits were more likely to be male, present lower total IQ scores (table 1), and experience trauma between ages 5 and 11 (only exception sociability; Supplementary Tables 1 & 2).

**Table 1.** Characteristics of Individuals With and Without Autistic Traits^a^

	Autism Factor Mean Score^b^ (*n* = 5800)			Social Communication ­Difficulties (*n* = 5106)			Repetitive Behaviors (*n* = 5127)			Pragmatic Language (*n* = 5086)			Sociability (*n* = 5434)		
Variable	Yes	No	*P*-value^c^	Yes	No	*P*-value^c^	Yes	No	*P*-value^c^	Yes	No	*P*-value^c^	Yes	No	*P*-value^c^
Total *n* (%)	457 (7.9)	5343 (92.1)	N/A	461 (9.0)	4645 (91.0)	N/A	313 (6.1)	4814 (93.9)	N/A	450 (8.9)	4636 (91.2)	N/A	600 (11.0)	4834 (89.0)	N/A
Male sex, *n* (%)	330 (72.2)	2571 (48.1)	<.001	298 (64.4)	2257 (48.6)	<.001	194 (62.0)	2377 (49.4)	<.001	284 (63.1)	2250 (48.5)	<.001	354 (59.0)	2379 (49.2)	<.001
Parity (<=1 child), *n* (%)	354 (77.5)	4449 (83.3)	.002	367 (79.6)	3888 (83.7)	.02	259 (82.8)	4011 (83.3)	.79	369 (82.0)	3873 (83.5)	.40	491 (81.8)	4024 (83.2)	.39
Maternal educational attainment (university degree), *n* (%)	70 (15.3)	904 (16.9)	.38	71 (15.4)	841 (18.1)	.15	59 (18.9)	836 (17.4)	.50	75 (16.7)	825 (17.8)	.55	91 (15.2)	834 (17.3)	.20
Mother’s age at delivery, mean (SD)	29.2 (4.6)	29.4 (4.4)	.51	29.2 (4.6)	29.5 (4.4)	.11	29.4 (4.5)	29.5 (4.4)	.60	29.5 (4.3)	29.5 (4.4)	.91	29.2 (4.3)	29.4 (4.4)	.33
Maternal depression during pregnancy (EPDS ≥ 12), *n* (%)	99 (21.7)	716 (13.4)	<.001	102 (22.1)	590 (12.7)	<.001	60 (19.2)	628 (13.1)	.002	90 (20.0)	604 (13.3)	<.001	88 (14.7)	649 (13.4)	.40
Total IQ score (WISC-III), mean (SD)	93.6 (18.1)	105.8 (15.9)	<.001	99.6 (19.1)	106.2 (15.9)	<.001	101.8 (18.4)	105.7 (16.2)	<.001	96.1 (17.9)	106.5 (15.8)	<.001	103.4 (15.9)	105.2 (16.5)	.01
Maternal anxiety during pregnancy, mean (SD)	5.4 (3.6)	4.5 (3.3)	<.001	5.4 (3.6)	4.48 (3.3)	<.001	5.7 (3.4)	4.5 (3.3)	<.001	5.2 (3.6)	4.5 (3.3)	<.001	4.6 (3.4)	4.6 (3.3)	.92
Major financial problems (present), *n* (%)	81 (17.7)	705 (13.2)	.01	91 (19.7)	575 (12.4)	<.001	48 (15.3)	614 (12.8)	.19	63 (14.0)	591 (12.8)	.45	79 (13.2)	642 (13.3)	.94

*Note:* SD, standard deviation; EPDS, Edinburgh Postnatal Depression Scale; IQ, Intelligence Quotient; WISC-III, Wechsler Intelligence Scale for Children third edition.

^a^Characteristics are shown for observations with complete data on exposure and confounders.

^b^Dichotomised (worst 10th percentile) for the purposes of the sample descriptive statistics.

^c^The *P*-values for *n* (%) and mean (SD) are based on Pearson χ^2^ test and independent-samples *t*-test, respectively.

Approximately 23%–25% of the sample had complete data on exposure, outcome, and confounders. Participants with complete data were more likely to be female, have a higher socioeconomic background, and present higher total IQ scores, while they were less likely to have experienced childhood trauma and psychotic experiences, compared to those with incomplete data (Supplementary Tables 3 & 4). After imputing data, the maximum sample size for our analyses was 13 105 individuals.

### Autistic Traits and Psychotic Experiences

As shown in table 2, there was evidence of associations between autism factor mean score and psychotic experiences (OR_CRUDE_ = 1.13, 95%CI 1.02–1.26, *P* = .03) as well as distressing and/or frequent psychotic experiences (OR_CRUDE_ = 1.20, 95%CI 1.04–1.38, *P* = .01). The associations remained of comparable magnitude when we adjusted for confounders (OR_ADJUSTED_ = 1.09, 95%CI 0.97–1.23, *P* = .15; OR_ADJUSTED_ = 1.19, 95%CI 1.01–1.39, *P* = .03) or schizophrenia polygenic risk (Supplementary Table 5), or restricted to psychotic experiences without tactile hallucinations (table 2).

**Table 2.** Associations Between Autistic Traits and Psychotic Experiences^a^

		Including Tactile Hallucinations								Excluding Tactile Hallucinations							
		Psychotic Experiences at Age 18/24				Psychotic Experiences at Age 18/24, ­Distressing and/or Frequent				Psychotic Experiences at Age 18/24				Psychotic Experiences at Age 18/24, Distressing and/or Frequent			
		Unadjusted		Adjusted^b^		Unadjusted		Adjusted^b^		Unadjusted		Adjusted^b^		Unadjusted		Adjusted^b^	
Exposure	*n*	OR (95%CI)	*P*-value	OR (95%CI)	*P*-value	OR (95%CI)	*P*-value	OR (95%CI)	*P*-value	OR (95%CI)	*P*-value	OR (95%CI)	*P*-value	OR (95%CI)	*P*-value	OR (95%CI)	*P*-value
Autism factor mean score	3707	1.13 (1.02–1.26)	.03	1.09 (0.97–1.23)	.15	1.20 (1.04–1.38)	.01	1.19 (1.01–1.39)	.03	1.15 (1.03–1.28)	.01	1.09 (0.96–1.23)	.17	1.18 (1.02–1.36)	.03	1.14 (0.97–1.35)	.11
Social communication difficulties	3384	1.43 (1.01–2.03)	.04	1.34 (0.94–1.91)	.11	1.60 (1.02–2.52)	.04	1.54 (0.97–2.45)	.07	1.49 (1.04–2.12)	.03	1.36 (0.95–1.96)	.10	1.69 (1.07–2.67)	.02	1.61 (1.01–2.56)	.05
Repetitive behavior	3397	0.98 (0.63–1.54)	.94	0.94 (0.60–1.48)	.78	1.17 (0.65–2.09)	.61	1.14 (0.64–2.06)	.66	0.98 (0.61–1.56)	.74	0.92 (0.58–1.48)	.74	1.13 (0.62–2.06)	.70	1.09 (0.59–2.01)	.78
Sociability	3536	1.28 (0.94–1.73)	.12	1.27 (0.94–1.73)	.12	1.31 (0.87–1.98)	.20	1.33 (0.88–2.02)	.18	1.25 (0.92–1.72)	.16	1.25 (0.91–1.71)	.18	1.20 (0.78–1.86)	.40	1.22 (0.79–1.88)	.38
Pragmatic language	3409	1.08 (0.75–1.55)	.68	1.00 (0.69–1.45)	.99	1.45 (0.92–2.28)	.11	1.37 (0.85–2.18)	.19	1.15 (0.80–1.66)	.45	1.04 (0.71–1.52)	.82	1.54 (0.98–2.42)	.06	1.43 (0.89–2.29)	.14

*Note:* OR, odds ratio; CI, confidence interval.

^a^Estimates based on observations with complete data on exposure, outcome, and confounders.

^b^Adjusted for child sex (male/female), parity (≤1 child versus ≥2 children), major financial problems in the family when the child was 8 months old (yes/no), maternal highest educational attainment, maternal age (at delivery), maternal Crown-Crisp anxiety scores (18 weeks gestation), maternal depression measured with the Edinburgh Postnatal Depression Scale (EPDS; 18 weeks gestation scores ≥ 13), and child IQ scores at age 8 assessed with the Wechsler Intelligence Scale for Children third edition (WISC-III).

Additionally, we found evidence of associations between social communication difficulties and psychotic experiences (OR_CRUDE_ = 1.43, 95%CI 1.01–2.03, *P* = .04) as well as distressing and/or frequent psychotic experiences (OR_CRUDE_ = 1.60, 95%CI 1.02–2.52, *P* = .04). Effect estimates were of comparable magnitude when we adjusted for confounders (OR_ADJUSTED_ = 1.34, 95%CI 0.94–1.91, *P* = .11; OR_ADJUSTED_ = 1.54, 95%CI 0.97–2.45, *P* = .07) or schizophrenia polygenic risk (Supplementary Table 5), or restricted to psychotic experiences without tactile hallucinations (table 2).

The imputed data analysis supported the identified associations (Supplementary Table 6), as estimates were of comparable magnitude to the primary analyses, and with higher precision.

There was less evidence of an association between repetitive behavior, pragmatic language, and sociability with any psychotic experiences measure (table 2). On this basis, we conducted sensitivity analyses under the scenario that the MAR assumption was completely violated, using social communication difficulties and autism factor mean score, considering that they were the exposures presenting the strongest associations with the outcome. Specifically, in a sample of *n* = 8106 participants with complete data on social communication difficulties, 3702 had missing data on the outcome and were recoded as having psychotic experiences (Supplementary Tables 7 & 8). Similarly, in a sample of *n* = 13 105 participants with complete data on autism factor mean score, 7600 participants had missing data on psychotic experiences and were recoded as having psychotic experiences (Supplementary Tables 9 & 10). Logistic regression analyses yielded confidence intervals that were overlapping, and in most cases completely bounded, across sensitivity, complete case, and imputed data analyses (Supplementary Tables 11 & 12).

### Mediation Analysis

The results of the mediation analyses are shown in table 3. Autism factor mean score, social communication difficulties, and psychotic experiences were associated with traumatic experiences at ages 5 to 11 (Supplementary Tables 1 & 2).

**Table 3.** Results of the Mediation Analyses With Childhood Trauma for the Associations Between Autism Mean Factor Score, Social Communication Difficulties, and Psychotic Experiences^a^

	Unadjusted		Adjusted^b^	
Estimate	OR (95%CI)	*P*-value	OR (95%CI)	*P*-value
*Exposure: Autism mean factor score; Outcome: psychotic experiences until age 24 (n* *=* *3577)*				
Natural direct effect	1.08 (0.97–1.21)	.18	1.06 (0.94–1.20)	.36
Natural indirect effect	1.06 (1.03–1.08)	<.001	1.04 (1.02–1.06)	<.001
Total effect	1.14 (1.02–1.28)	.02	1.10 (0.97–1.25)	.14
Proportion mediated	45%		41%	
*Exposure: Autism mean factor score; Outcome: psychotic experiences until age 24 distressing/frequent (n* *=* *3577)*				
Natural direct effect	1.15 (0.98–1.35)	.10	1.15 (0.96–1.37)	.12
Natural indirect effect	1.07 (1.04–1.10)	<.001	1.05 (1.02–1.07)	<.001
Total effect	1.23 (1.04–1.44)	.01	1.20 (1.01–1.44)	.04
Proportion mediated	35%		28%	
*Exposure: Social communication difficulties; Outcome: psychotic experiences until age 24 (n* *=* *3326)*				
Natural direct effect	1.27 (0.90–1.80)	.17	1.22 (0.86–1.73)	.26
Natural indirect effect	1.15 (1.08–1.22)	<.001	1.11 (1.05–1.18)	<.001
Total effect	1.46 (1.03–2.06)	.03	1.36 (0.96–1.92)	.08
Proportion mediated	41%		38%	
*Exposure: Social communication difficulties; Outcome: psychotic experiences until age 24 distressing/frequent (n = 3326)*				
Natural direct effect	1.38 (0.87–2.18)	.17	1.37 (0.87–2.15)	.18
Natural indirect effect	1.18 (1.09–1.27)	<.001	1.15 (1.06–1.23)	<.001
Total effect	1.62 (1.03–2.55)	.04	1.57 (1.00–2.45)	.05
Proportion mediated	40%		36%	

^a^Estimates based on observations with complete data on exposure, mediator, outcome, and confounders.

^b^Adjusted for the following confounders: child sex, parity, major financial problems, maternal highest educational attainment, maternal anxiety, maternal depression, and child IQ.

There was evidence to suggest that the associations between autism factor mean score and psychotic experiences were mediated by childhood traumatic experiences in crude and adjusted for confounder models (NIE OR_CRUDE_ = 1.06, 95%CI 1.03–1.08, *P* < .001, PM = 45%; NIE OR_ADJUSTED_ = 1.04, 95%CI 1.02–1.06, *P* < .001, PM = 41%). Analyses with distressing and/or frequent psychotic experiences yielded comparable natural indirect effect estimates (NIE OR_CRUDE_ = 1.07, 95%CI 1.04–1.1, *P* < .001, PM = 35%; NIE OR_ADJUSTED_ = 1.05, 95%CI 1.02–1.07, *P* < .001, PM = 28%).

Additionally, we found evidence consistent with a mediating effect of childhood traumatic experiences in the associations between social communication difficulties and psychotic experiences in crude and adjusted models (NIE OR_CRUDE_ = 1.15, 95%CI 1.08–1.22, *P* < .001, PM = 41%; NIE OR_ADJUSTED_ = 1.11, 95%CI 1.05–1.18, *P* < .001, PM = 38%). Comparable natural indirect effect estimates were identified in analyses assessing distressing and/or frequent psychotic experiences (NIE OR_CRUDE_ = 1.18, 95%CI 1.09–1.27, *P* < .001, PM = 40%; NIE OR_ADJUSTED_ = 1.15, 95%CI 1.06–1.23, *P* < .001, PM = 36%).

Results of the mediation analyses were similar when we assessed associations with psychotic experiences excluding tactile hallucinations, adjusted for schizophrenia PRS, or imputed missing data (Supplementary Tables 13–15).

## Discussion

Using population-based birth cohort data, we examined the association between autistic traits in childhood and psychotic experiences in adulthood, and the potential mediating role of traumatic experiences. We found that broad autistic traits, as captured by autism factor mean score, and social communication difficulties were associated with psychotic experiences up to age 24. There was limited evidence to suggest associations between measures of repetitive behavior, pragmatic language, or sociability and psychotic experiences. The relationship between autism factor mean score, social communication difficulties, and psychotic experiences was substantially mediated by traumatic experiences in early childhood, and not confounded by schizophrenia PRS.

Our longitudinal study is the first to show a relationship between childhood autistic traits and psychotic experiences up to age 24 years. It extends two previous ALSPAC studies which found that psychotic experiences at age 12 years were more common in autistic children or children with autistic traits.^4,5^ However, these studies also observed associations with measures of repetitive behaviors and pragmatic language.^5^ Two other cohort studies observed weaker or no evidence for a relationship between autistic traits and psychotic experiences, with one study reporting only modest correlations between autistic traits at ages 8–16 years and psychotic experiences at age 16 years,^7^ and the other reporting weak associations between autistic traits at ages 9 or 12 years and psychotic experiences at ages 15 or 18 years.^6^ One possibility is that varying operationalizations of autistic traits might account for discrepant results. For instance, children with social communication difficulties might be especially prone to developing persistent psychotic experiences because they encounter more problems in social relationships than individuals exhibiting repetitive behaviors, and consequently, factors such as increased feelings of isolation, distrust, and defeat might make those individuals more vulnerable to developing psychosis.^51,52^

Among children scoring positively on the measures of autism mean factor score or social communication difficulties, point estimates for the occurrence of distressing and/or frequent psychotic experiences in early adulthood were particularly high. Notably, three previous studies have also shown that (for as of yet unknown reasons) autistic individuals reported more distress when experiencing psychotic symptoms than non-autistic peers.^9,53,54^ These distressing and/or frequent psychotic experiences appear to be most strongly related to negative mental health consequences, such as the development of psychotic disorder.^38^ Still, thus far there has been little work examining how these psychotic experiences can be best identified and addressed in individuals with autistic traits, and more work is needed in this area.

The association between autistic traits and psychotic experiences was not strongly influenced by schizophrenia PRS, but substantially mediated by interpersonal trauma in childhood. These findings are consistent with the idea that the association between autism and psychosis is influenced by environmental factors and not the sole result of a shared genetic liability. The experience of trauma in childhood is a well-established risk factor for psychosis.^15,55^ However, despite reports of elevated rates of trauma in autistic individuals or individuals with autistic traits,^22,56^ studies of its mental health consequences are lacking.^57^ Our findings indicate that trauma may be an important, potentially modifiable pathway between autistic features and later onset of psychotic experiences, and more work is necessary to examine how (the consequences of) trauma can best be prevented, identified, or treated in autistic individuals. For instance, there is early work showing that eye movement desensitization and reprocessing (EMDR) can be safely and effectively used among individuals with a psychotic disorder,^58^ and its efficacy for autistic individuals with psychotic symptoms could be assessed. Additionally, elucidating the mechanisms through which traumatic experiences lead to psychosis, building on work in nonautistic populations,^52,55,59^ can be an important avenue for future research.

Of note, with regards to the causal pathways tested in this study, there was a partial overlap in the ages at which autistic traits and childhood trauma were measured. This exposure-mediator overlap might preclude strong causal inferences, as autistic-like traits might have been exacerbated by exposure to traumatic events. Indeed, detrimental effects of childhood adversity on social cognitive functioning have been reported.^60^ However, it is worth noting that in the ALSPAC cohort, social communication difficulties are associated with autism PRS, suggesting developmental origins.^24^ In addition, social communication difficulties in the ALSPAC cohort seem to be relatively stable over time for male as well as female participants.^3^ These studies support the idea that particularly social communication difficulties measured in the context of the present study do not necessarily stem from trauma exposure alone but reflect autism-related difficulties.

Strengths of our study include its longitudinal design and long-term follow-up in a general population-based cohort. The study also has limitations. First, our complete-records analysis might have been influenced by a lower statistical power and/or selection bias due to attrition. However, analyses using imputed data yielded comparable results to the complete-records analyses. Second, multiple imputation is a widely used approach to address missing data, but it presents important pitfalls that should be acknowledged. The most important is that the method requires the MAR assumption to hold. Since the MAR assumption is not directly testable the possibility of biased estimates cannot be excluded. However, we followed established guidelines to include auxiliary variables and make the MAR assumption more plausible, while in addition, we performed sensitivity analyses to test the association estimates in the extreme scenario that the MAR assumption was completely violated. Third, a substantial number of models were run to examine the association between autistic traits and psychotic experiences, which could increase the likelihood of false-positive findings. However, it is important to note the consistency of the association estimates across analyses and that the vast majority of the tests conducted were conducted in order to test the robustness of our findings and overcome limitations of previous studies investigating psychotic outcomes. Fourth, traumatic experiences were measured with a combination of parental- and self-reports. Parents are likely to underreport the occurrence of traumatic events, whereas retrospective self-reports might overestimate (due to recall bias) or underestimate (due to nondifferential measurement error) trauma prevalence. Finally, as in every observational analysis, the possibility of residual confounding cannot be excluded.

Future studies are expected to further elucidate present findings. Specifically, the increasing availability of large multi-ethnic ancestry samples with extensive information on childhood neurodevelopment, life events, and adulthood psychopathology can provide valuable insights into the complex relationship between autism and psychosis. A particularly promising avenue for research would be the investigation of whether specific trauma types mediate the pathways linking autistic traits to a range of adverse mental health outcomes, including negative and positive psychotic symptoms, depressive symptoms, and anxiety.

In conclusion, broad autistic traits and especially social communication difficulties in childhood appear to be associated with psychotic experiences until young adulthood. This association is unlikely explained by genetic risk as captured by current schizophrenia PRS. Childhood trauma constitutes a potentially modifiable environmental risk factor for psychosis in autistic individuals that warrants further attention in research and clinical practice.

## Supplementary Material

sbac167_suppl_Supplementary_Material
