# Supplementary material for: Childhood Trauma As a Mediator of the Association Between Autistic Traits and Psychotic Experiences: Evidence From the Avon Longitudinal Study of Parents and Children Cohort
Source: Schizophr Bull. 2022 Nov 26;49(2):364–74. doi: 10.1093/schbul/sbac167 (PMC10016398; doi:10.1093/schbul/sbac167)
Supplement: sbac167_suppl_Supplementary_Material [file sbac167_suppl_supplementary_material.docx]

**Supplementary Material**

**Table of contents**

[**Supplementary Figure 1.** Available sample sizes for the primary analyses. 2](#_Toc116565874)

[**Supplementary Methods 1.** Schizophrenia polygenic risk score (PRS) calculation. 3](#_Toc116565875)

[**Supplementary Methods 2.** Auxiliary variables used for multiple imputation. 4](#_Toc116565876)

[**Supplementary Table 1.** Associations between autistic traits and childhood traumatic experiences^1^. 5](#_Toc116565877)

[**Supplementary Table 2.** Prevalence of traumatic experiences by autism factor mean score, social communication difficulties, and psychotic experiences for each sample used in the mediation analyses. 6](#_Toc116565878)

[**Supplementary Table 3.** Summary of the characteristics for the sample with complete records across each analysis and the ALSPAC sample. 7](#_Toc116565879)

[**Supplementary Table 4.** Predictors of being a complete case^1^ across each analysis. 8](#_Toc116565880)

[**Supplementary Table 5.** Association between autistic traits and psychotic experiences, adjusted for schizophrenia polygenic risk scores (PRS)^1^. 9](#_Toc116565881)

[**Supplementary Table 6.** Associations between autistic traits and psychotic experiences using 100 imputed datasets. 10](#_Toc116565882)

[**Supplementary Table 7.** Summary of data on psychotic experiences in the sample with complete data on social communication difficulties. 11](#_Toc116565883)

[**Supplementary Table 8.** Summary of data on psychotic experiences after recoding participants with missing data as having psychotic experiences. 11](#_Toc116565884)

[**Supplementary Table 9.** Summary of data on psychotic experiences in the sample with complete data on autism factor mean score. 11](#_Toc116565885)

[**Supplementary Table 10.** Summary of data on psychotic experiences after recoding participants with missing data as having psychotic experiences. 11](#_Toc116565886)

[**Supplementary Table 11.** Associations between social communication difficulties and psychotic experiences in complete case analyses, imputed data analyses and scenario that participants with missing data presented psychotic experiences. 12](#_Toc116565887)

[**Supplementary Table 12.** Associations between autism factor mean score with psychotic experiences in complete case analyses, imputed data analyses and scenario that participants with missing data presented psychotic experiences. 12](#_Toc116565888)

[**Supplementary Table 13.** Mediation analyses results for the associations between autism factor mean score and social communication difficulties with psychotic experiences, excluding tactile hallucinations. 13](#_Toc116565889)

[**Supplementary Table 14.** Mediation analyses results for the associations between autism factor mean score and social communication difficulties with psychotic experiences adjusting for schizophrenia polygenic risk scores (PRS)^1^. 14](#_Toc116565890)

[**Supplementary Table 15.** Mediation analyses results for the associations between autism factor mean score and social communication difficulties with psychotic experiences using imputed data. 15](#_Toc116565891)

[**Supplementary references** 16](#_Toc116565892)

# **Supplementary Figure 1.** Available sample sizes for the primary analyses.


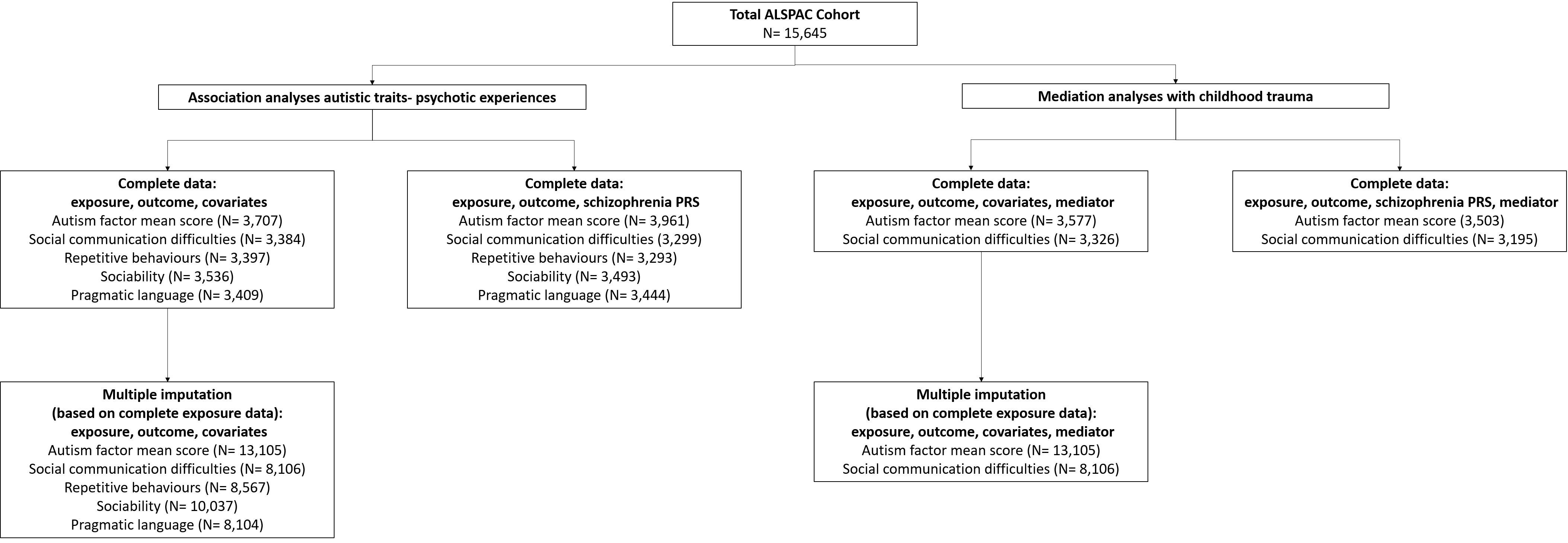


# **Supplementary Methods 1.** Schizophrenia polygenic risk score (PRS) calculation.

PRSs were calculated using PLINK version 1.9, applying the method described by the Psychiatric Genomics Consortium^1^.

A total of 9,912 ALSPAC children were genotyped on the Illumina HumanHap550-quad. After standard quality control (details on quality control in the ALSPAC cohort can be found elsewhere^2^) and excluding participants who had withdrawn consent, genetic data were available for 7,977 children of European ancestry.

Using the latest European ancestry genome-wide association study (GWAS) summary data for schizophrenia^1^ as discovery sample, we extracted single nucleotide polymorphisms (SNPs), corresponding alleles, effect sizes and *p*-values. SNPs with mismatching alleles between the discovery and the ALSPAC genotyped sample were removed. We additionally removed the MHC region (25 Mb – 34 Mb), except for one SNP representing the strongest signal within the region. Using ALSPAC data as reference panel, SNPs were clumped with an *r*^2^ of 0.25 and a physical distance threshold of 500 kB.

We calculated PRS for each participant across 13 *p*-value thresholds (5e-8 to 0.5), standardized by subtracting the mean and dividing by the standard deviation.

# **Supplementary Methods 2.** Auxiliary variables used for multiple imputation.

Following guidelines detailed in Lee et al.^3^, in our imputation models we included exposure, outcome, mediator, confounders and three auxiliary variables. The decision was based not only on their potential associations with exposure, outcome, mediator, confounders, but also on their completeness. Specifically:

1. Maternal marital status: assessed via questionnaire during 8 weeks of gestation. The measure was available in 13,545 mothers (13% missingness).
2. Home ownership status: assessed via questionnaire during 8 weeks of gestation. The measure was available in 13,487 mothers (14% missingness).
3. Crowding index: assessed via questionnaire during 8 weeks of gestation and defined as the proportion of people per room. The measure was available in 13,247 mothers (15% missingness).

# **Supplementary Table 1.** Associations between autistic traits and childhood traumatic experiences^1^.

|  |  | Traumatic experiences between ages 5-11 | | | |
| --- | --- | --- | --- | --- | --- |
| Exposure | *n* | Unadjusted | | Adjusted^2^ | |
|  |  | OR  (95% CIs) | *p*-value | OR  (95% CIs) | *p*-value |
| Autism mean factor score | 5,438 | 1.41  (1.32–1.50) | <0.001 | 1.28  (1.20–1.38) | <0.001 |
| Social communication difficulties | 4,959 | 2.54  (2.08–3.11) | <0.001 | 2.20  (1.79–2.70) | <0.001 |
| Repetitive behaviours | 5,036 | 1.57  (1.25–1.98) | <0.001 | 1.35  (1.07–1.71) | 0.013 |
| Sociability | 5,210 | 0.97  (0.82–1.16) | 0.76 | 0.93  (0.78–1.11) | 0.415 |
| Pragmatic language | 4,946 | 1.82  (1.50–2.22) | <0.001 | 1.57  (1.28–1.92) | <0.001 |
| OR, odds ratio; CI, confidence interval.  ^1^ Estimates based on observations with complete data on exposure, confounders, and childhood trauma.  ^2^ Adjusted for the following confounders: child sex, parity, major financial problems, maternal highest educational attainment, maternal anxiety, maternal depression, and child IQ. | | | | | |

| \| **Supplementary Table 2.** Prevalence of traumatic experiences by autism factor mean score, social communication difficulties, and psychotic experiences for each sample used in the mediation analyses. \| \| \| \| \| \| \| \| \| \| --- \| --- \| --- \| --- \| --- \| --- \| --- \| --- \| --- \| \|  \| Complete  records^1^  *n* = 3,577 \| \| \| \| Complete  records^1^  *n* = 3,326 \| \| \| \| \|  \| Exposure:  Autism factor mean score^2^ \| \| Outcome:  Psychotic experiences \| \| Exposure:  Social communication difficulties \| \| Outcome:  Psychotic experiences \| \| \| Variable \| No^3^  *n* (%) \| Yes^4^  *n* (%) \| No^5^  *n* (%) \| Yes^6^  *n* (%) \| No^3^  *n* (%) \| Yes^4^  *n* (%) \| No^5^  *n* (%) \| Yes^6^  *n* (%) \| \| Experienced childhood trauma between ages 5-11 years \| 1,288  (38.83%) \| 160  (61.54%) \| 1,220  (38.77%) \| 228  (53.02%) \| 1,154  (37.66%) \| 168  (64.12%) \| 1,118  (38.17%) \| 204  (51.39%) \| \| Did not experience childhood trauma between ages 5-11 years \| 2,029  (61.17%) \| 100  (38.46%) \| 1,927  (61.23%) \| 202  (46.98%) \| 1,910  (62.34%) \| 94  (35.88%) \| 1,811  (61.83%) \| 193  (48.61%) \| \| ^1^ Sample with complete data on exposure, mediator, confounders, and outcome.  ^2^ The measure was dichotomised for the purposes of sample descriptive statistics.  ^3^ Child has score in the lower 90^th^ percentile.  ^4^ Child has score in the upper decile.  ^5^ Psychotic experiences not present until age 24.  ^6^ Psychotic experiences present until age 24. \| \| \| \| \| \| \| \| \| |
| --- | --- | --- | --- | --- | --- | --- | --- | --- | --- | --- | --- | --- | --- | --- | --- | --- | --- | --- | --- | --- | --- | --- | --- | --- | --- | --- | --- | --- | --- | --- | --- | --- | --- | --- | --- | --- | --- | --- | --- | --- | --- | --- | --- | --- | --- | --- | --- | --- | --- | --- | --- | --- | --- | --- | --- | --- | --- | --- | --- | --- | --- | --- | --- |

| **Supplementary Table 3.** Summary of the characteristics for the sample with complete records across each analysis and the ALSPAC sample. | | | | | | |
| --- | --- | --- | --- | --- | --- | --- |
|  | Exposure in each analysis | | | | |  |
|  | Autism factor mean score | Social communication difficulties | Repetitive behaviours | Sociability | Pragmatic language |  |
| Variable | Complete  records^1^  *n* = 3,707 | Complete  records^1^  *n* = 3,384 | Complete records^1^  *n* =3,397 | Complete records^1^  *n* =3,536 | Complete  records^1^  *n* = 3,409 | Full sample^2^  *n* = 14,868 |
| Male sex, *n* (%) | 1,649 (44%) | 1,514 (45%) | 1,529 (45%) | 1,589 (45%) | 1,518 (45%) | 7,591 (51%) |
| Parity (<=1 child), *n* (%) | 3,138 (85%) | 2,861 (85%) | 2,880 (85%) | 3,000 (85%) | 2,891 (85%) | 10,295 (80%) |
| Maternal educational attainment (university degree), *n* (%) | 761 (21%) | 726 (21%) | 711 (21%) | 730 (21%) | 723 (21%) | 1,598 (13%) |
| Major financial problems (present), *n* (%) | 462 (12%) | 406 (12%) | 404 (12%) | 437 (12%) | 411 (12%) | 1,665 (15%) |
| Maternal depression during pregnancy (EPDS >= 12), *n* (%) | 468 (13%) | 410 (12%) | 420 (12%) | 439 (12%) | 426 (13%) | 2,122 (18%) |
| Mother’s age at delivery, mean (SD) | 30 (4.4) | 30 (4.3) | 30 (4.4) | 30 (4.4) | 30 (4.4) | 28 (4.9) |
| Maternal anxiety during pregnancy, mean (SD) | 4.5 (3.3) | 4.5 (3.3) | 4.5 (3.3) | 4.5 (3.3) | 4.5 (3.3) | 4.9 (3.6) |
| Total IQ score (WISC-III), mean (SD) | 107 (16.1) | 108 (15.9) | 108 (16.0) | 108 (16.0) | 108 (16.1) | 104 (16.5) |
| Psychotic experiences until 24, *n* (%) | 448 (12%) | 404 (12%) | 411 (12%) | 429 (12%) | 408 (12%) | 770 (13%) |
| Traumatic experiences 5-11 years, *n* (%) | 1,448 (40%) | 1,322 (40%) | 1,346 (40%) | 1,382 (40%) | 1,334 (40%) | 3,658 (42%) |
| Autism factor mean score, *n* (%)^3^ | 268 (7%) | 234 (7%) | 241 (7%) | 251 (7%) | 249 (7%) | 1,309 (10%) |
| SD, standard deviation; EPDS, Edinburgh Postnatal Depression Scale; IQ, Intelligence Quotient; WISC-III, Wechsler Intelligence Scale for Children third edition.  ^1^ Sample with complete data on exposure, outcome, confounders.  ^2^ ALSPAC children alive at 1 year and not withdrawn consent. Different completion rates across each variable.  ^3^ The measure was dichotomised (worst 10^th^ percentile) for the purposes of sample descriptive statistics. | | | | | | |

| **Supplementary Table 4.** Predictors of being a complete case^1^ across each analysis. | | | | | | | | | | |
| --- | --- | --- | --- | --- | --- | --- | --- | --- | --- | --- |
|  | Exposure in each analysis | | | | | | | | | |
|  | Autism factor mean score | | Social communication difficulties | | Repetitive behaviours | | Sociability | | Pragmatic language | |
| Predictor variable | OR (95% CIs) | *p*-value | OR (95% CIs) | *p*-value | OR (95% CIs) | *p*-value | OR (95% CIs) | *p*-value | OR (95% CIs) | *p*-value |
| Sex (female) | 1.42  (1.32–1.53) | <0.001 | 1.39  (1.29–1.5) | <0.001 | 1.37  (1.27–1.48) | <0.001 | 1.38  (1.28–1.49) | <0.001 | 1.41  (1.30–1.52) | <0.001 |
| Parity (>1 child) | 0.63  (0.57–0.70) | <0.001 | 0.65  (0.58–0.72) | <0.001 | 0.63  (0.57–0.70) | <0.001 | 0.63  (0.56–0.69) | <0.001 | 0.63  (0.57–0.70) | <0.001 |
| Maternal educational attainment (university degree) | 2.42  (2.18–2.70) | <0.001 | 2.54  (2.28–2.83) | <0.001 | 2.42  (2.17–2.69) | <0.001 | 2.39  (2.15–2.66) | <0.001 | 2.49  (2.24–2.77) | <0.001 |
| Major financial problems (present) | 0.76  (0.67–0.85) | <0.001 | 0.72  (0.64–0.81) | <0.001 | 0.71  (0.63–0.80) | <0.001 | 0.75  (0.67–0.84) | <0.001 | 0.73  (0.64–0.82) | <0.001 |
| Maternal depression during pregnancy (EPDS >= 12) | 0.58  (0.52–0.64) | <0.001 | 0.55  (0.49–0.62) | <0.001 | 0.56  (0.50–0.63) | <0.001 | 0.56  (0.50–0.63) | <0.001 | 0.57  (0.51–0.64) | <0.001 |
| Psychotic experiences until 24 (present) | 0.75  (0.64–0.87) | <0.001 | 0.75  (0.64–0.87) | <0.001 | 0.78  (0.67–0.90) | 0.001 | 0.77  (0.66–0.90) | 0.001 | 0.75  (0.65–0.88) | <0.001 |
| Traumatic experiences 5-11 years | 0.89  (0.82–0.97) | 0.01 | 0.85  (0.78–0.93) | <0.001 | 0.87  (0.79–0.95) | 0.002 | 0.87  (0.79–0.94) | 0.001 | 0.86  (0.79–0.94) | 0.001 |
| Autism factor mean score (above worst 10^th^ percentile)^2^ | 0.62  (0.54–0.72) | <0.001 | 0.59  (0.51–0.69) | <0.001 | 0.62  (0.53–0.71) | <0.001 | 0.61  (0.53–0.71) | <0.001 | 0.64  (0.55–0.74) | <0.001 |
| Mother’s age at delivery (per year increase) | 1.10  (1.09–1.11) | <0.001 | 1.11  (1.09–1.12) | <0.001 | 1.10  (1.10–1.11) | <0.001 | 1.10  (1.09–1.11) | <0.001 | 1.10  (1.10–1.11) | <0.001 |
| Maternal anxiety during pregnancy, (per point increase) | 0.95  (0.94–0.96) | <0.001 | 0.95  (0.94–0.96) | <0.001 | 0.95  (0.94–0.96) | <0.001 | 0.95  (0.94–0.96) | <0.001 | 0.95  (0.94–0.96) | <0.001 |
| Total IQ score (WISC-III), (per point increase) | 1.03  (1.02–1.03) | <0.001 | 1.03  (1.02–1.03) | <0.001 | 1.03  (1.02–1.03) | <0.001 | 1.03  (1.02–1.03) | <0.001 | 1.03  (1.02–1.03) | <0.001 |
| ^1^ Sample with complete data on exposure, outcome, and confounders.  ^2^ The measure was dichotomised (worst 10^th^ percentile) for the purposes of sample descriptive statistics. | | | | | | | | | | |

# **Supplementary Table 5.** Association between autistic traits and psychotic experiences, adjusted for schizophrenia polygenic risk scores (PRS)^1^.

|  | | Including tactile hallucinations | | | | | | | | Excluding tactile hallucinations | | | | | | | |
| --- | --- | --- | --- | --- | --- | --- | --- | --- | --- | --- | --- | --- | --- | --- | --- | --- | --- |
|  |  | Psychotic experiences at age 18/24 | | | | Psychotic experiences at age 18/24, distressing and/or frequent | | | | Psychotic experiences at age 18/24 | | | | Psychotic experiences at age 18/24, distressing and/or frequent | | | |
|  |  | Unadjusted | | Adjusted^2^ | | Unadjusted | | Adjusted^2^ | | Unadjusted | | Adjusted^2^ | | Unadjusted | | Adjusted^2^ | |
| Exposure | *n* | OR  (95% CI) | *p*-value | OR  (95% CI) | *p*-value | OR  (95% CI) | *p*-value | OR  (95% CI) | *p*-value | OR  (95% CI) | *p*-value | OR  (95% CI) | *p*-value | OR  (95% CI) | *p*-value | OR  (95% CI) | *p*-value |
| Autism factor mean score | 3,961 | 1.10  (0.99–1.22) | 0.07 | 1.10  (0.99–1.21) | 0.08 | 1.15  (1.01–1.32) | 0.03 | 1.15  (1.01–1.32) | 0.04 | 1.14  (1.02–1.26) | 0.02 | 1.13  (1.02–1.26) | 0.02 | 1.17  (1.02–1.33) | 0.03 | 1.17  (1.02–1.33) | 0.03 |
| Social communication difficulties | 3,299 | 1.47  (1.04–2.08) | 0.03 | 1.46  (1.03–2.07) | 0.03 | 1.68  (1.07–2.62) | 0.02 | 1.68  (1.07–2.62) | 0.02 | 1.46  (1.01–2.08) | 0.05 | 1.44  (1.00–2.08) | 0.05 | 1.59  (1.00–2.53) | 0.05 | 1.59  (1.00–2.53) | 0.05 |
| Repetitive behaviour | 3,293 | 0.80  (0.50–1.29) | 0.37 | 0.80  (0.50–1.29) | 0.36 | 1.00  (0.55–1.82) | 0.99 | 1.00  (0.55–1.83) | 0.99 | 0.85  (0.52–1.39) | 0.52 | 0.85  (0.52–1.39) | 0.52 | 0.96  (0.51–1.79) | 0.89 | 0.96  (0.51–1.80) | 0.90 |
| Sociability | 3,493 | 1.13  (0.83–1.54) | 0.45 | 1.13  (0.83–1.54) | 0.45 | 1.18  (0.78–1.79) | 0.44 | 1.18  (0.78–1.79) | 0.45 | 1.09  (0.79–1.51) | 0.60 | 1.09  (0.79–1.51) | 0.60 | 1.03  (0.66–1.61) | 0.89 | 1.03  (0.66–1.61) | 0.89 |
| Pragmatic language | 3,444 | 1.12  (0.79–1.59) | 0.51 | 1.12  (0.79–1.58) | 0.52 | 1.43  (0.92–2.21) | 0.11 | 1.43  (0.92–2.21) | 0.11 | 1.23  (0.87–1.75) | 0.24 | 1.23  (0.87–1.75) | 0.24 | 1.52  (0.98–2.35) | 0.06 | 1.52  (0.98–2.35) | 0.06 |
| OR, odds ratio; CI, confidence interval.  ^1^ Estimates based on observations with complete data on exposure, outcome, and schizophrenia polygenic risk scores.  ^2^ Adjusted for schizophrenia polygenic risk scores. | | | | | | | | | | | | | | | | | |

# **Supplementary Table 6.** Associations between autistic traits and psychotic experiences using 100 imputed datasets.

|  | | Including tactile hallucinations | | | | | | | | Excluding tactile hallucinations | | | | | | | |
| --- | --- | --- | --- | --- | --- | --- | --- | --- | --- | --- | --- | --- | --- | --- | --- | --- | --- |
|  |  | Psychotic experiences at age 18/24 | | | | Psychotic experiences at age 18/24, distressing and/or frequent | | | | Psychotic experiences at age 18/24 | | | | Psychotic experiences at age 18/24, distressing and/or frequent | | | |
|  |  | Unadjusted | | Adjusted^1^ | | Unadjusted | | Adjusted^1^ | | Unadjusted | | Adjusted^1^ | | Unadjusted | | Adjusted^1^ | |
| Exposure | *n* | OR  (95% CI) | *p*-value | OR  (95% CI) | *p*-value | OR  (95% CI) | *p*-value | OR  (95% CI) | *p*-value | OR  (95% CI) | *p*-value | OR  (95% CI) | *p*-value | OR  (95% CI) | *p*-value | OR  (95% CI) | *p*-value |
| Autism factor mean score | 13,105 | 1.11  (1.02–1.20) | 0.02 | 1.06  (0.97–1.17) | 0.20 | 1.15  (1.03–1.28) | 0.01 | 1.11  (0.98–1.26) | 0.07 | 1.13  (1.04–1.23) | 0.005 | 1.08  (0.98–1.19) | 0.13 | 1.14  (1.02–1.28) | 0.02 | 1.09  (0.96–1.24) | 0.16 |
| Social communication difficulties | 8,106 | 1.42  (1.05–1.92) | 0.02 | 1.32  (0.97–1.08) | 0.07 | 1.73  (1.18–2.54) | 0.006 | 1.62  (1.09–2.41) | 0.02 | 1.46  (1.07–1.99) | 0.02 | 1.33  (0.97–1.83) | 0.08 | 1.69  (1.14–2.51) | 0.01 | 1.57  (1.04–2.37) | 0.03 |
| Repetitive behaviour | 8,567 | 1.16  (0.81–1.64) | 0.41 | 1.07  (0.75–1.53) | 0.71 | 1.34  (0.85–2.11) | 0.21 | 1.25  (0.79–2.00) | 0.34 | 1.20  (0.83–1.73) | 0.33 | 1.10  (0.75–1.60) | 0.62 | 1.32  (0.82–2.13) | 0.25 | 1.22  (0.75–2.01) | 0.42 |
| Sociability | 10,037 | 1.20  (0.92–1.55) | 0.17 | 1.16  (0.89–1.52) | 0.26 | 1.27  (0.89–1.8) | 0.19 | 1.23  (0.86–1.77) | 0.26 | 1.17  (0.89–1.54) | 0.27 | 1.13  (0.86–1.50) | 0.38 | 1.14  (0.79–1.65) | 0.48 | 1.10  (0.76–1.60) | 0.61 |
| Pragmatic language | 8,104 | 1.16  (0.86–1.57) | 0.32 | 1.07  (0.78–1.46) | 0.68 | 1.26  (0.85–1.87) | 0.24 | 1.14  (0.77–1.71) | 0.53 | 1.23  (0.91–1.67) | 0.17 | 1.11  (0.80–1.52) | 0.54 | 1.34  (0.91–1.97) | 0.14 | 1.20  (0.80–1.80) | 0.38 |
| OR, odds ratio; CI, confidence interval.  ^1^ Adjusted for child sex (male/female), parity (≤ 1 child versus ≥ 2 children), major financial problems in the family when the child was 8 months old (yes/no), maternal highest educational attainment, maternal age (at delivery), maternal Crown-Crisp anxiety scores (18 weeks gestation), maternal depression measured with the Edinburgh Postnatal Depression Scale (EPDS; 18 weeks gestation scores ≥ 13), and child IQ scores at age 8 assessed with the Wechsler Intelligence Scale for Children third edition (WISC-III). | | | | | | | | | | | | | | | | | |

# **Supplementary Table 7.** Summary of data on psychotic experiences in the sample with complete data on social communication difficulties.

|  | Psychotic experiences at age 18/24 | | |  |
| --- | --- | --- | --- | --- |
| Social communication difficulties | Without psychotic experiences | With psychotic experiences | Missing data  on psychotic experiences | Total |
| Without difficulties | 3,558 | 494 | 3,256 | 7,308 |
| With difficulties | 295 | 57 | 446 | 798 |
| Total | 3,853 | 551 | 3,702 | 8,106 |

# **Supplementary Table 8.** Summary of data on psychotic experiences after recoding participants with missing data as having psychotic experiences.

|  | Psychotic experiences at age 18/24 | |  |
| --- | --- | --- | --- |
| Social communication difficulties | Without psychotic experiences | With psychotic experiences | Total |
| Without difficulties | 3,558 | 3,750 | 7,308 |
| With difficulties | 295 | 503 | 798 |
| Total | 3,853 | 4,253 | 8,106 |

# **Supplementary Table 9.** Summary of data on psychotic experiences in the sample with complete data on autism factor mean score.

|  | Psychotic experiences at age 18/24 | | |  |
| --- | --- | --- | --- | --- |
| Autism factor mean score^1^ | Without psychotic experiences | With psychotic experiences | Missing data  on psychotic experiences | Total |
| Without difficulties | 4,418 | 645 | 6,731 | 11,794 |
| With difficulties | 366 | 76 | 869 | 1,311 |
| Total | 4,784 | 721 | 7,600 | 13,105 |
| ^1^ Dichotomised (worst 10th percentile) for the purposes of the sample summary data. | | | | |

# **Supplementary Table 10.** Summary of data on psychotic experiences after recoding participants with missing data as having psychotic experiences.

|  | Psychotic experiences at age 18/24 | |  |
| --- | --- | --- | --- |
| Autism factor mean score^1^ | Without psychotic experiences | With psychotic experiences | Total |
| Without difficulties | 4,418 | 7,376 | 11,794 |
| With difficulties | 366 | 945 | 1,311 |
| Total | 4,784 | 8,321 | 13,105 |
| ^1^ Dichotomised (worst 10th percentile) for the purposes of the sample summary data. | | | |

# **Supplementary Table 11.** Associations between social communication difficulties and psychotic experiences in complete case analyses, imputed data analyses and scenario that participants with missing data presented psychotic experiences.

|  |  | Psychotic experiences at age 18/24 | |
| --- | --- | --- | --- |
|  |  | Unadjusted^1^ | Adjusted^2^ |
| Analysis: | *n* | OR (95% CIs) | OR (95% CIs) |
| Complete case | 3,384 | 1.43 (1.01–2.03) | 1.34 (0.94–1.91) |
| Imputed data | 8,106 | 1.42 (1.05–1.92) | 1.32 (0.97–1.08) |
| Sensitivity analysis^3^ | 8,106 | 1.62 (1.39–1.88) | 1.24 (1.05–1.45) |
| ^1^ Crude analyses.  ^2^ Analyses adjusted for child sex (male/female), parity (≤ 1 child versus ≥ 2 children), major financial problems in the family when the child was 8 months old (yes/no), maternal highest educational attainment, maternal age (at delivery), maternal Crown-Crisp anxiety scores (18 weeks gestation), maternal depression measured with the Edinburgh Postnatal Depression Scale (EPDS; 18 weeks gestation scores ≥ 13), and child IQ scores at age 8 assessed with the Wechsler Intelligence Scale for Children third edition (WISC-III).  ^3^ Sensitivity analysis in which all participants with missing data were recoded as having psychotic experiences. | | | |

# **Supplementary Table 12.** Associations between autism factor mean score with psychotic experiences in complete case analyses, imputed data analyses and scenario that participants with missing data presented psychotic experiences.

|  |  | Psychotic experiences at age 18/24 | |
| --- | --- | --- | --- |
|  |  | Unadjusted^1^ | Adjusted^2^ |
| Analysis: | *n* | OR (95% CIs) | OR (95% CIs) |
| Complete case | 3,707 | 1.13 (1.02–1.26) | 1.09 (0.97–1.23) |
| Imputed data | 13,105 | 1.11 (1.02–1.20) | 1.06 (0.97–1.17) |
| Sensitivity analysis^3^ | 13,105 | 1.27 (1.22–1.32) | 1.00 (0.96–1.05) |
| ^1^ Crude analyses.  ^2^ Analyses adjusted for child sex (male/female), parity (≤ 1 child versus ≥ 2 children), major financial problems in the family when the child was 8 months old (yes/no), maternal highest educational attainment, maternal age (at delivery), maternal Crown-Crisp anxiety scores (18 weeks gestation), maternal depression measured with the Edinburgh Postnatal Depression Scale (EPDS; 18 weeks gestation scores ≥ 13), and child IQ scores at age 8 assessed with the Wechsler Intelligence Scale for Children third edition (WISC-III).  ^3^ Sensitivity analysis in which all participants with missing data were recoded as having psychotic experiences. | | | |

# **Supplementary Table 13.** Mediation analyses results for the associations between autism factor mean score and social communication difficulties with psychotic experiences, excluding tactile hallucinations.

|  | Unadjusted | | Adjusted^2^ | |
| --- | --- | --- | --- | --- |
| Estimate | OR (95% CI) | *p*-value | OR (95% CI) | *p*-value |
| *Exposure: Autism mean factor score; Outcome: psychotic experiences until age 24 (n = 3,577)* |  |  |  |  |
| Natural direct effect | 1.11 (0.99–1.24) | 0.08 | 1.07 (0.94–1.22) | 0.28 |
| Natural indirect effect | 1.06 (1.03–1.08) | <0.001 | 1.04 (1.02–1.06) | <0.001 |
| Total effect | 1.17 (1.04–1.31) | 0.01 | 1.11 (0.98–1.26) | 0.11 |
| Proportion mediated | 38% | | 38% |  |
|  |  | |  |  |
| *Exposure: Autism mean factor score; Outcome: psychotic experiences until age 24 distressing/frequent (n = 3,577)* |  |  |  |  |
| Natural direct effect | 1.12 (0.95–1.33) | 0.18 | 1.10 (0.92–1.33) | 0.29 |
| Natural indirect effect | 1.07 (1.04–1.10) | <0.001 | 1.05 (1.02–1.07) | <0.001 |
| Total effect | 1.21 (1.01–1.43) | 0.03 | 1.16 (0.96–1.40) | 0.12 |
| Proportion mediated | 40% | | 35% | |
|  |  | |  | |
| *Exposure: Social communication difficulties; Outcome: psychotic experiences until age 24 (n = 3,326)* |  |  |  |  |
| Natural direct effect | 1.33 (0.93–1.89) | 0.12 | 1.25 (0.88–1.78) | 0.21 |
| Natural indirect effect | 1.14 (1.07–1.21) | <0.001 | 1.10 (1.04–1.17) | 0.001 |
| Total effect | 1.51 (1.06–2.15) | 0.02 | 1.38 (0.98–1.96) | 0.07 |
| Proportion mediated | 36% | | 33% | |
|  |  | |  | |
| *Exposure: Social communication difficulties; Outcome: psychotic experiences until age 24 distressing/frequent (n = 3,326)* |  |  |  |  |
| Natural direct effect | 1.45 (0.92–2.30) | 0.11 | 1.43 (0.90–2.25) | 0.13 |
| Natural indirect effect | 1.18 (1.09–1.28) | <0.001 | 1.15 (1.07–1.24) | <0.001 |
| Total effect | 1.72 (1.09–2.70) | 0.02 | 1.64 (1.05–2.57) | 0.03 |
| Proportion mediated | 37% | | 33% | |
| OR, odds ratio; CI, confidence interval.  ^1^ Estimates based on observations with complete data on exposure, mediator, outcome, and confounders.  ^2^ Adjusted for the following confounders: child sex, parity, major financial problems, maternal highest educational attainment, maternal anxiety, maternal depression, and child IQ. | | | | |

| **Supplementary Table 14.** Mediation analyses results for the associations between autism factor mean score and social communication difficulties with psychotic experiences adjusting for schizophrenia polygenic risk scores (PRS)^1^. | | | | | | | | |
| --- | --- | --- | --- | --- | --- | --- | --- | --- |
|  | | | | |  | | | |
|  | Unadjusted | | Adjusted^2^ | | Unadjusted | | Adjusted^2^ | |
| Estimate | OR (95% CI) | *p*-value | OR (95% CI) | *p*-value | OR (95% CI) | *p*-value | OR (95% CI) | *p*-value |
|  | *Exposure: Autism mean factor score; Outcome: psychotic experiences until age 24 (n = 3,503)* | | | | *Exposure: Autism mean factor score; Outcome: psychotic experiences until age 24 excluding tactile hallucinations (n = 3,503)* | | | |
| Natural direct effect | 1.04 (0.93–1.16) | 0.47 | 1.04 (0.93–1.16) | 0.46 | 1.08 (0.97–1.21) | 0.15 | 1.08 (0.97–1.21) | 0.14 |
| Natural indirect effect | 1.06 (1.03–1.08) | <0.001 | 1.06 (1.03–1.08) | <0.001 | 1.06 (1.04–1.08) | <0.001 | 1.06 (1.04–1.08) | <0.001 |
| Total effect | 1.10 (0.98–1.23) | 0.09 | 1.10 (0.98–1.23) | 0.09 | 1.15 (1.03–1.28) | 0.02 | 1.15 (1.03–1.28) | 0.01 |
| Proportion mediated | 61% | | 61% | | 45% | | 42% | |
|  | | | | |  | | | |
|  | *Exposure: Autism mean factor score; Outcome: psychotic experiences until age 24 distressing/frequent (n = 3,503)* | | | | *Exposure: Autism mean factor score; Outcome: psychotic experiences until age 24 distressing/frequent excluding tactile hallucinations*  *(n = 3,503)* | | | |
| Natural direct effect | 1.08 (0.93–1.26) | 0.33 | 1.08 (0.93–1.26) | 0.30 | 1.09 (0.93–1.27) | 0.27 | 1.09 (0.94–1.28) | 0.25 |
| Natural indirect effect | 1.06 (1.03–1.09) | <0.001 | 1.06 (1.04–1.09) | <0.001 | 1.06 (1.03–1.09) | <0.001 | 1.06 (1.03–1.09) | <0.001 |
| Total effect | 1.15 (0.98–1.34) | 0.08 | 1.15 (0.98–1.34) | 0.07 | 1.16 (0.99–1.36) | 0.06 | 1.16 (0.99–1.36) | 0.06 |
| Proportion mediated | 45% | | 42% | | 42% | | 40% | |
|  | | | | |  | | | |
|  | *Exposure: Social communication difficulties; Outcome: psychotic experiences until age 24 (n = 3,195)* | | | | *Exposure: Social communication difficulties; Outcome: psychotic experiences until age 24 excluding tactile hallucinations (n = 3,195)* | | | |
| Natural direct effect | 1.28 (0.90–1.82) | 0.16 | 1.28 (0.90–1.81) | 0.16 | 1.24 (0.86–1.80) | 0.24 | 1.24 (0.86–1.80) | 0.23 |
| Natural indirect effect | 1.14 (1.08–1.21) | <0.001 | 1.14 (1.08–1.21) | <0.001 | 1.15 (1.08–1.22) | <0.001 | 1.15 (1.08–1.22) | <0.001 |
| Total effect | 1.40 (1.03–2.08) | 0.04 | 1.40 (1.02–2.07) | 0.03 | 1.43 (0.99–2.08) | 0.06 | 1.43 (0.98–2.07) | 0.06 |
| Proportion mediated | 39% | | 39% | | 44% | | 43% | |
|  | | | | |  | | | |
|  | *Exposure: Social communication difficulties; Outcome: psychotic experiences until age 24 distressing/frequent (n = 3,195)* | | | | *Exposure: Social communication difficulties; Outcome: psychotic experiences until age 24 distressing/frequent excluding tactile hallucinations (n = 3,195)* | | | |
| Natural direct effect | 1.40 (0.88–2.25) | 0.16 | 1.41 (0.88–2.25) | 0.15 | 1.33 (0.82–2.17) | 0.25 | 1.33 (0.82–2.17) | 0.24 |
| Natural indirect effect | 1.16 (1.08–1.25) | <0.001 | 1.16 (1.08–1.25) | <0.001 | 1.16 (1.08–1.25) | <0.001 | 1.16 (1.08–1.25) | <0.001 |
| Total effect | 1.63 (1.02–2.62) | 0.04 | 1.63 (1.02–2.62) | 0.04 | 1.55 (0.94–2.53) | 0.08 | 1.55 (0.94–2.54) | 0.08 |
| Proportion mediated | 36% | | 35% | | 39% | | 39% | |
| OR, odds ratio; CI, confidence interval.  ^1^ Estimates based on observations with complete data on exposure, mediator, outcome, and schizophrenia polygenic risk scores.  ^2^ Adjusted for schizophrenia polygenic risk scores. | | | | | | | | |

# **Supplementary Table 15.** Mediation analyses results for the associations between autism factor mean score and social communication difficulties with psychotic experiences using imputed data.

|  | Including tactile hallucinations | | | | | | | | Excluding tactile hallucinations | | | | | | | |
| --- | --- | --- | --- | --- | --- | --- | --- | --- | --- | --- | --- | --- | --- | --- | --- | --- |
|  | Psychotic experiences at age 18/24 | | | | Psychotic experiences at age 18/24, distressing and/or frequent | | | | Psychotic experiences at age 18/24 | | | | Psychotic experiences at age 18/24, distressing and/or frequent | | | |
|  | Unadjusted | | Adjusted^1^ | | Unadjusted | | Adjusted^1^ | | Unadjusted | | Adjusted^1^ | | Unadjusted | | Adjusted^1^ | |
| Estimate | OR  (95% CI) | *p*-value | OR  (95% CI) | *p*-value | OR  (95% CI) | *p*-value | OR  (95% CI) | *p*-value | OR  (95% CI) | *p*-value | OR  (95% CI) | *p*-value | OR  (95% CI) | *p*-value | OR  (95% CI) | *p*-value |
| *Exposure: autism factor mean score (n = 13,105)* |  | | | | | | | | | | | | | | | |
| Natural direct effect | 1.08  (1.01–1.16) | 0.02 | 1.02  (0.96–2.13) | 0.55 | 1.15  (1.07–1.23) | <0.001 | 1.10  (0.96–1.26) | 0.18 | 1.10  (1.02–1.17) | 0.01 | 1.06  (0.95–1.17) | 0.30 | 1.12  (1.05–1.21) | 0.001 | 1.08  (0.94–1.24) | 0.30 |
| Natural indirect effect | 1.06  (1.05–2.18) | <0.001 | 1.02  (1.01–1.29) | <0.001 | 1.09  (1.07–1.12) | <0.001 | 1.02  (1.01–1.48) | 0.001 | 1.07  (1.05–2.24) | <0.001 | 1.02  (1.01–1.39) | <0.001 | 1.08  (1.06–2.57) | <0.001 | 1.03  (1.01–1.51) | 0.001 |
| Total effect | 1.15  (1.07–1.23) | <0.001 | 1.03  (0.98–1.10) | 0.26 | 1.26  (1.17–1.35) | <0.001 | 1.12  (0.98–1.29) | 0.09 | 1.17  (1.09–1.25) | <0.001 | 1.08  (0.97–1.20) | 0.15 | 1.21  (1.13–1.30) | <0.001 | 1.11  (0.96–1.27) | 0.16 |
| Proportion mediated | 45% | | 51% | | 41% | | 18% | | 44% | | 26% | | 43% | | 29% | |
|  |  | |  | |  | |  | |  | |  | |  | |  | |
| *Exposure: social communication difficulties (n = 8,106)* |  | | | | | | | | | | | | | | | |
| Natural direct effect | 1.13  (0.84–1.53) | 0.43 | 1.13  (0.81–1.58) | 0.47 | 1.16  (0.82–1.63) | 0.41 | 1.41  (0.94–2.10) | 0.10 | 1.16  (0.86–1.58) | 0.33 | 1.16  (0.83–1.62) | 0.39 | 1.14  (0.81–1.62) | 0.45 | 1.38  (0.91–2.08) | 0.13 |
| Natural indirect effect | 1.16  (1.11–1.21) | <0.001 | 1.10  (1.05–1.15) | <0.001 | 1.18  (1.12–1.24) | <0.001 | 1.14  (1.08–1.19) | <0.001 | 1.16  (1.11–1.22) | <0.001 | 1.10  (1.06–1.15) | <0.001 | 1.18  (1.12–1.25) | <0.001 | 1.14  (1.08–1.20) | <0.001 |
| Total effect | 1.31  (0.97–1.77) | 0.08 | 1.24  (0.89–1.73) | 0.20 | 1.36  (0.97–1.93) | 0.08 | 1.60  (1.07–2.38) | 0.022 | 1.35  (1.00–1.84) | 0.05 | 1.28  (0.91–1.79) | 0.15 | 1.35  (0.96–1.90) | 0.08 | 1.58  (1.05–2.37) | 0.03 |
| Proportion mediated | 58% | | 47% | | 57% | | 32% | | 54% | | 42% | | 59% | | 34% | |
| OR, odds ratio; CI, confidence interval.  ^1^ Adjusted for child sex (male/female), parity (≤ 1 child versus ≥ 2 children), major financial problems in the family when the child was 8 months old (yes/no), maternal highest educational attainment, maternal age (at delivery), maternal Crown-Crisp anxiety scores (18 weeks gestation), maternal depression measured with the Edinburgh Postnatal Depression Scale (EPDS; 18 weeks gestation scores ≥ 13), and child IQ scores at age 8 assessed with the Wechsler Intelligence Scale for Children third edition (WISC-III). | | | | | | | | | | | | | | | | |

# **Supplementary references**

1. Trubetskoy V, Pardiñas AF, Qi T, et al. Mapping genomic loci implicates genes and synaptic biology in schizophrenia. *Nature.* 2022;604(7906):502-508.

2. Stergiakouli E, Gaillard R, Tavaré JM, et al. Genome‐wide association study of height‐adjusted BMI in childhood identifies functional variant in ADCY3. *Obesity.* 2014;22(10):2252-2259.

3. Lee KJ, Tilling KM, Cornish RP, et al. Framework for the treatment and reporting of missing data in observational studies: The Treatment And Reporting of Missing data in Observational Studies framework. *J Clin Epidemiol.* 2021;134:79-88.
